# Postoperative Changes in Femoral Rotation Angle and Their Influencing Factors Following Total Hip Arthroplasty via Single Approach: A Retrospective CT-Based Study

**DOI:** 10.3390/jcm15072729

**Published:** 2026-04-04

**Authors:** Hiroaki Kurishima, Yasutake Tomata, Norikazu Yamada, Atsushi Noro, Yasuaki Kuriyama, Hidetatsu Tanaka, Yu Mori, Toshimi Aizawa

**Affiliations:** 1 Department of Orthopaedic Surgery, Japanese Red Cross Sendai Hospital, 2-43-3 Yagiyamahoncho, Taihaku-Ku, Sendai 982-8501, Miyagi, Japan; 2 Department of Orthopaedic Surgery, Tohoku University Graduate School of Medicine, 1-1 Seiryo-Machi, Aoba-Ku, Sendai 980-8574, Miyagi, Japan; 3 Faculty of Health and Social Service, Kanagawa University of Human Services, 1-10-1 Heiseicho, Yokosuka 238-8522, Kanagawa, Japan

**Keywords:** total hip arthroplasty, femoral rotation angle, femoral anteversion, optimal stem anteversion, multivariate regression analysis

## Abstract

**Background/Objectives:** This study aimed to evaluate the femoral rotation angle (FRA) before and after THA using a single approach and to identify its influencing factors through three-dimensional measurements. **Methods:** This retrospective study analyzed patients undergoing 108 primary unilateral THA via the anterolateral-supine approach (ALSA) from May to October 2023. Patients with hip contractures, femoral deformities, or other specific conditions were excluded for precise FRA measurements. Preoperative and postoperative CT scans were used for measurements of the FRA, anteversion, leg lengthening, and global offset. FRA was defined as the angle between the posterior condylar line and the line connecting the bilateral anterosuperior iliac spines, with external rotation as positive. Multiple linear regression, adjusted for age, sex, body mass index, and stem design, assessed the influence of anteversion change, leg lengthening, global offset change, and soft tissue release on the difference in FRA. **Results:** The mean FRA changed significantly from −2.8° preoperatively to −11.8° postoperatively (*p* < 0.001), demonstrating an average internal rotation of 9.0° after THA. Anteversion increased by a mean of 9.0° (*p* < 0.001), leg length increased by 9.0 mm (*p* < 0.001), and global offset decreased by 1.7 mm (*p* < 0.001). Multivariate analysis revealed that anteversion change (β = −0.41, *p* < 0.001) and global offset change (β = 0.40, *p* = 0.022) were significantly associated with FRA differences. Leg lengthening and ischiofemoral ligament or conjoint tendon resection were not significant (*p* = 0.089, *p* = 0.917, and *p* = 0.750, respectively). **Conclusions:** ALSA THA significantly rotates the femur internally, associated with an increase in anteversion and a decrease in global offset.

## 1. Introduction

Complications of total hip arthroplasty (THA), particularly dislocation, remain a major concern and sometimes require revision surgery [1]. Various strategies have been explored to reduce the risk of dislocation, including the use of larger femoral heads [2], minimally invasive surgery [3,4], and the combined anteversion theory [5,6,7]. Pelvic motion has been increasingly recognized as a critical factor in preventing dislocations, with numerous studies investigating alterations in pelvic tilt across different patient demographics [8] and functional positions [9,10]. Similarly to pelvic motion, femoral rotation varies among individuals and changes with body positions [11,12]. This implies that functional anteversion must also be considered to prevent dislocations [12,13]. However, studies comparing preoperative and postoperative femoral rotation angle (FRA) and its contributing factors remain limited [11,13,14,15,16]. To prevent dislocation, further study on the factors influencing differences in FRA is necessary.

Although previous studies have consistently reported that the femur rotates internally after THA [11,13,14,15,16], standardized muscle-sparing surgical approaches and precise measurements of key parameters—including anteversion, leg lengthening, and global offset—are essential to eliminate surgical variability and achieve a true three-dimensional evaluation. However, previous studies have limitations, such as mixing the anterior approach (preserving muscles) and posterior approach (requiring muscle resection), relying predominantly on two-dimensional radiographic measurements, or lacking certain parameters. To establish a more comprehensive framework for optimizing stem anteversion and femoral alignment, further research using high-precision measurement techniques that consider soft tissue balance is required.

To improve the accuracy of the analysis regarding the relationship between FRA and various factors, this study exclusively included patients who underwent THA using the muscle-sparing anterolateral-supine approach (ALSA), while also accounting for any minor soft tissue releases performed during the procedures. Furthermore, using computed tomography (CT), we three-dimensionally assessed how changes in femoral anteversion, leg length, and global offset affected the postoperative FRA. Our hypothesis was that our precise measurement method would identify the factors influencing the changes in the FRA before and after THA.

## 2. Materials and Methods

### 2.1. Study Design and Setting

This retrospective cohort study was approved by the Institutional Review Board of Japanese Red Cross Sendai Hospital (approval number, 2023-01; approval date, 14 February 2023) and informed consent was obtained from all participants. Data collected from a single high-volume institution between May 2023 and October 2023 were utilized.

### 2.2. Study Participants

We reviewed consecutive patients who underwent primary THA using the ALSA. In cases where a patient underwent THA on both hips during the study period, only one side was randomly selected to ensure data independence. Patients were excluded based on the following criteria: (1) unavailable CT data; (2) hip contractures (defined as an inability to achieve 0° of flexion/extension or abduction/adduction); (3) preoperative femoral deformities or fractures; (4) intraoperative or postoperative fractures; (5) ipsilateral total knee arthroplasty or marked femoral neck shortening; and (6) the use of a stem design in fewer than five cases.

### 2.3. Surgical Methods

All procedures were performed by three experienced orthopedic surgeons (NY, AN, and HK) using the ALSA. The surgical methodology has been previously documented [17,18]. The anterior capsule was resected in a reverse T-shape with preservation of the medial iliofemoral ligament and was repaired during closure. In cases where femoral broach insertion was difficult, the ischiofemoral ligament (ISFL) and conjoint tendon were resected stepwise. The stems used during this study period were primarily cementless stems, consisting of two main design types: tapered wedge designs and fully hydroxyapatite-coated designs. Stem selection was determined at the surgeon’s discretion, targeting an anteversion corresponding to the native angle. If the anteversion exceeded 50° or was below 10°, modifications were made to achieve an anteversion range of 10° to 50°. Postoperatively, all patients followed a standardized rehabilitation protocol. Full weight-bearing was permitted immediately as tolerated, and no specific activity or movement restrictions were imposed. Patients typically practiced walking and muscle strengthening with a physical therapist during their hospital stay (approximately 1–2 weeks) and were discharged once they achieved independent mobility.

### 2.4. Data Collection

**Figure 1:**
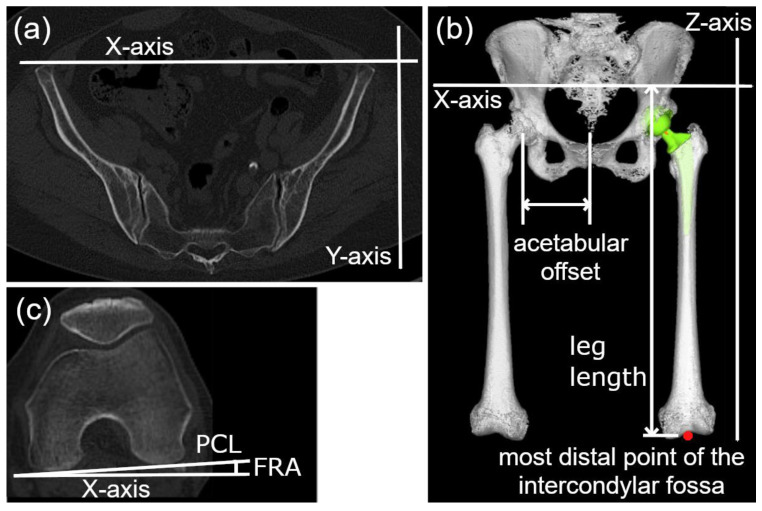
Definitions of the floor pelvic plane (**a**,**b**), the acetabular offset (**b**), the most distal point of the intercondylar fossa (indicated by the red point), and the posterior condylar line (**c**). PCL: posterior condylar line; FRA: femoral rotation angle.

Preoperative CT scans were obtained one month before surgery, and postoperative scans were taken one to four months after surgery. The legs were positioned in each patient’s physiologically neutral position. According to the previous report [11,13,14,15,16], measurements for FRA, leg lengthening, global offset change, and anteversion were conducted using three-dimensional preoperative and postoperative planning software for THA (ZedHip version 19.0.1.0, LEXI Co., Tokyo, Japan). Anatomical landmarks were initially identified through software-assisted automated algorithms and were subsequently manually verified and adjusted by the observers in multi-planar reconstruction views to ensure precise identification. The functional pelvic plane (FPP) was defined using a line connecting the bilateral anterosuperior iliac spines along the X-axis [19]. The Y-axis was set perpendicular to the FPP, and the Z-axis was oriented orthogonally to the X- and Y-axes (Figure 1a,b) [19]. FRA was defined as the angular measurement between the posterior condylar line (PCL) and the X-axis, with external rotation designated as positive (Figure 1c) [12]. Leg lengthening was calculated using the preoperative and postoperative Z-axis values of the most distal point of the intercondylar fossa after aligning the leg to the neutral hip position (Figure 1b).

**Figure 2:**
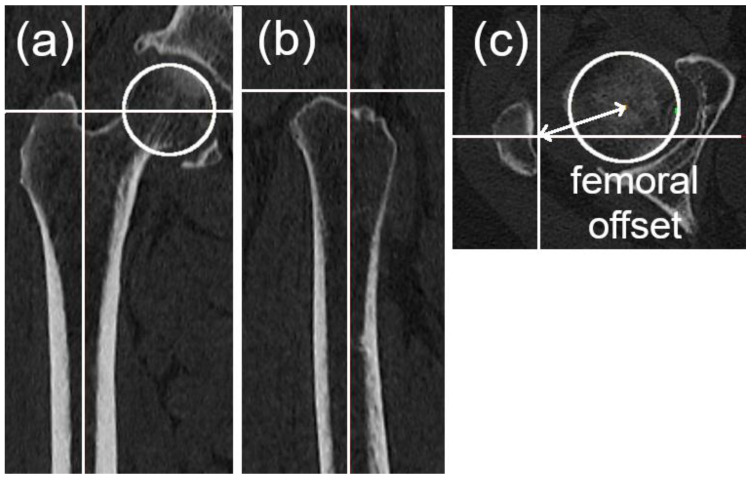
Definition of femoral offset. (**a**,**b**) The longitudinal axis of the proximal femur. (**c**) Femoral offset is defined as the distance (arrow) from the center of the femoral head (circle) to the longitudinal axis of the proximal femur. The horizontal and vertical lines in all panels represent the cross-reference lines used for alignment and measurement.

Global offset changes were determined by the difference in three-dimensional global offset measurements preoperatively and postoperatively [20]. The global offset was defined as the sum of the acetabular offset (distance from the femoral head center to the pubic symphysis plane) (Figure 1b) and femoral offset (distance from the femoral head center to the longitudinal axis of the proximal femur) (Figure 2) [20,21].

**Figure 3:**
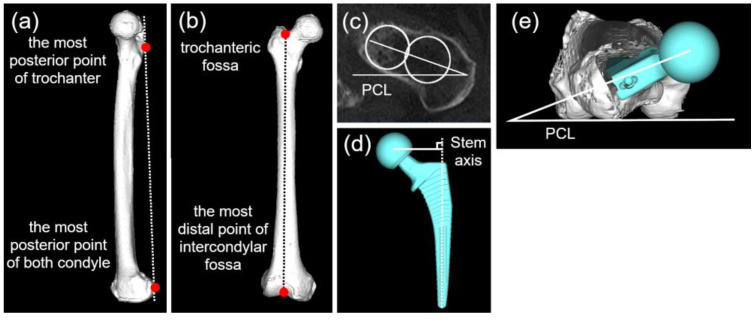
Definitions of the retrocondylar plane (**a**,**b**), preoperative anteversion (**c**), and postoperative anteversion (**d**,**e**). Red points in (**a**,**b**) indicate the anatomical landmarks labeled in each panel. The circles in (**c**) are used to identify the centers of the femoral neck for measurement. PCL: posterior condylar line.

Anteversion was measured using the femoral retrocondylar plane, defined by the posterior margin of the greater trochanter and both femoral posterior condyles (Figure 3a,b) [22]. The femoral Z-axis was the projected axis of the posterior condylar plane of the line connecting the trochanteric fossa and the most distal point of the intercondylar fossa (Figure 3b) [23]. Native femoral anteversion was defined as the angle between the neck centerline and the PCL at the most proximal portion of the femoral neck, excluding the femoral head, following Sugano’s method (Figure 3c) [23]. Postoperative anteversion was defined as a perpendicular line from the head center to the stem axis and PCL (Figure 3d,e).

### 2.5. Outcomes

The outcome was the change in the FRA from preoperative to postoperative assessments and its influencing factors. This was calculated as the difference between postoperative and preoperative FRA values (ΔFRA = postoperative FRA − preoperative FRA).

### 2.6. Data Analysis

The normality of the dependent variables and pre- and postoperative parameters (the FRA, anteversion, leg length and global offset) was assessed using the Kolmogorov–Smirnov test. Preoperative and postoperative FRA and other parameters were compared using a paired *t*-test. For the FRA measurements, the main outcome of this study, we assessed intra- and interobserver intraclass correlation coefficients (ICCs) in a randomly selected subset of 15 cases. This reliability analysis was performed by two independent observers (HK and YK), both of whom are orthopedic surgeons. To evaluate intra-observer reliability, Observer HK repeated the measurements twice for these 15 cases, with an interval of at least four weeks between sessions to minimize recall bias. Inter-observer reliability was determined by comparing the first set of measurements from Observer HK with those from Observer YK. Both observers were blinded to their previous measurements and to the patients’ clinical data. Intra- and interobserver reliability were evaluated using ICC (3,1) and ICC (2,1), respectively.

Multiple linear regression analysis was performed to examine the associations between exposure and outcome variables. The dependent variable was ΔFRA. Explanatory variables included anteversion change (°), leg lengthening (mm), global offset change (mm), and resection of the ISFL or conjoint tendon. Adjustment variables included sex, age, body mass index (BMI), and stem design based on prior research and clinical relevance. To evaluate the stability of the associations and account for confounding factors, a hierarchical regression analysis (Models 1 through 3) was employed. In Model 1, multivariable regression analyses were performed, adjusted for sex and age, with the explanatory variables [11,15]. Model 2 further included BMI and stem design to account for their potential influences on femoral rotation as surgery-related factors [24,25]. Finally, in Model 3, age, sex, body mass index, stem design and all explanatory factors were simultaneously entered into the model to evaluate their independent associations with changes in the FRA.

The model fit was evaluated using the coefficient of determination (R^2^), and multicollinearity was assessed using variance inflation factors (VIFs).

Statistical significance was set at *p* < 0.05, and analyses were performed using IBM SPSS Statistics version 30 (SPSS Inc., Chicago, IL, USA) and R version 4.5.3 (R Core Team, 2026) [26].

## 3. Results

### 3.1. Study Cohort Characteristics and Surgical Interventions

**Figure 4:**
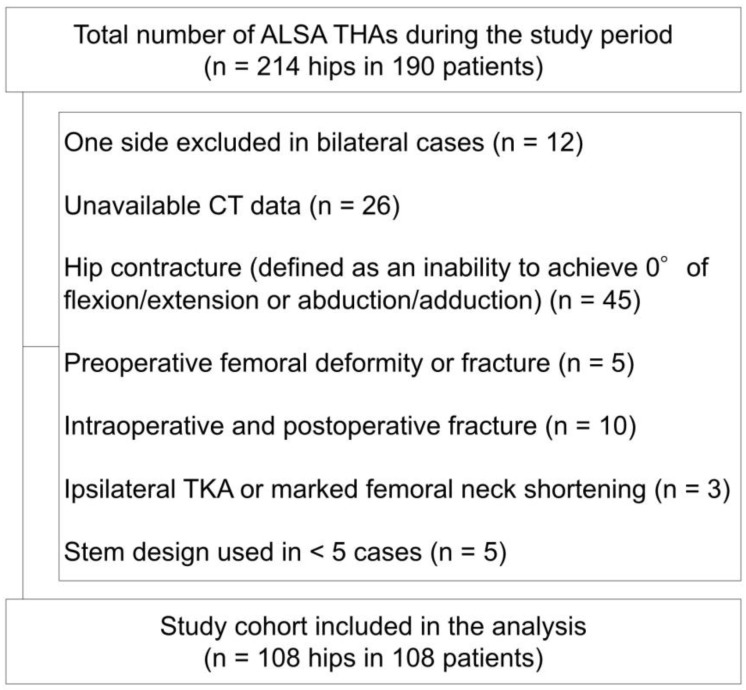
Flow diagram of study participants.

Between May 2023 and October 2023, a total of 214 primary THAs were performed using the ALSA in 190 patients. Following the exclusion of 106 hips based on the predefined criteria, the final study cohort consisted of 108 hips in 108 patients (Figure 4). Detailed patient demographics and surgical characteristics are summarized in Table 1. Regarding soft tissue management during surgery, the ISFL was resected in 80 hips (74.1%), and the conjoint tendon was resected in 34 hips (31.5%). None of the patients experienced dislocation.

### 3.2. Femoral Rotation Angle Changes Post-THA

The Kolmogorov–Smirnov test did not reject the normality of the outcome variables (*p* = 0.200).

The reliability analysis demonstrated excellent intra- and interobserver ICCs for the FRA measurements, with all ICCs exceeding 0.99 (Supplementary Table S1). Following THA, paired *t*-tests revealed a significant change in the mean FRA from −2.8° (range, −28.7 to 22.0) preoperatively to −11.8° (range, −48.3 to 16.2) postoperatively (*p* < 0.001). The mean rotation difference was −9.0° (range, −49.3 to 19.6), indicating an average internal femoral rotation of 9.0° post-THA. Among the 108 hips evaluated, 93 (86.1%) demonstrated internal rotation, with 46 (42.6%) exhibiting internal rotation exceeding 10°.

### 3.3. Changes in Anteversion, Leg Length, and Global Offset

Pre- and postoperative parameters, including anteversion, leg length, and global offset, all followed a normal distribution. The mean anteversion significantly increased from 22.6° (range, −20.0 to 53.2) to 31.6° (range, −3.6 to 62.5) (*p* < 0.001), representing a mean change of 9.0° (range, −10.9 to 32.3). Leg length increased in all cases, with a mean increment of 9.0 mm (range, 0.8 to 22.6; *p* < 0.001). In contrast, global offset measurements showed a mean reduction of 1.7 mm (range, −18.7 to 12.4; *p* < 0.001), with a decrease in global offset observed in 64 hips (59.3%).

### 3.4. Factors Influencing Postoperative Femoral Rotation Angle

In the final multivariate regression model (Model 3), preoperative to postoperative FRA differences demonstrated significant independent associations with anteversion change (β = −0.41, *p* < 0.001) and global offset change (β = 0.40, *p* = 0.022) (Table 2). In contrast, no significant associations were observed for leg lengthening (*p* = 0.089) and resection of the ISFL (*p* = 0.917) or resection of the ISFL and conjoint tendon (*p* = 0.750). The R^2^ value for Model 3 was 0.24. Furthermore, all VIFs remained below 5 across all models, confirming the absence of significant multicollinearity.

## 4. Discussion

This study revealed a significant change in the FRA after THA using ALSA, a muscle-sparing technique. The mean change was −9.0° (*p* < 0.001), indicating that ALSA THA frequently resulted in internal femoral rotation. Furthermore, changes in the FRA following THA were significantly associated with variations in anteversion, leg lengthening, and global offset changes. These findings were obtained through precise and reproducible methods using a single muscle-sparing approach and 3D-CT analysis.

Consistent with our findings, prior studies have reported that the femur rotates internally after THA [11,13,14,15,16]. While anteversion change was identified as an influencing factor for the FRA in these studies [14,15,16], the impact of global offset has not been established. To clarify the hypothesized but unproven relationship between global offset change and the FRA [13,16], we conducted a comprehensive evaluation using a standardized muscle-sparing approach and 3D-CT analysis. To our knowledge, this is the first report to demonstrate that global offset change is a significant independent factor associated with FRA change.

Regarding sex-related differences, we utilized pre- to postoperative “change” parameters as our primary outcomes and explanatory factors. Because these parameters reflect the magnitude of change rather than absolute values, the impact of baseline anatomical variations between sexes was likely minimized.

The biomechanical mechanisms underlying the relationships between FRA differences and changes in anteversion, leg length, and global offset may be hypothesized to involve muscle function and tension. Increased anteversion may relax the external rotator muscles at the posterior aspect of the femur, potentially facilitating internal rotation [16]. Leg lengthening and global offset changes had opposite associations with FRA change; however, these associations might be related to the direction of the external and internal rotators around the hip. Although the external rotator muscles run horizontally in the hip joint, there are no primary internal rotator muscles with a purely horizontal orientation. Instead, several secondary internal rotators run longitudinally, including the anterior fibers of the gluteus minimus and medius, tensor fasciae latae, adductor longus, adductor brevis, and pectineus [27]. In the postoperative period, the femur may undergo compensatory rotation to reach a new state of soft tissue equilibrium, where muscle tension is minimized. While leg lengthening increases tension in longitudinally oriented muscles, potentially leading to internal rotation, an increase in global offset tightens horizontally oriented muscles, which may lead to external rotation. These anatomical orientations of the periarticular muscles could potentially explain why leg lengthening and global offset changes showed opposite associations with FRA change in our study. However, as muscle and soft tissue tension were not directly quantified in this study, these biomechanical mechanisms remain speculative. Further studies, including musculoskeletal modeling or direct tension measurements, are warranted to clarify these potential causal relationships.

Furthermore, postoperative femoral rotation may be influenced by overall surgical component positioning, including the femoral stem and acetabular cup. In the present study, we addressed this by evaluating parameters such as “anteversion change” and “global offset change,” which reflect the composite result of cup position and stem alignment. Our multivariate analysis demonstrated that both anteversion change and global offset change were significant independent factors associated with FRA change.

Based on our findings, excessive changes in anteversion may be avoided. Theoretically, an equal but opposite change in the FRA could compensate for anteversion changes, thereby maintaining the impingement risk. However, our regression coefficient of anteversion on FRA difference was only −0.41 (95% confidence interval: −0.61, −0.21), indicating that a larger anteversion change increases the likelihood of impingement. In particular, an excessive decrease in anteversion seems more problematic than an excessive increase in preventing dislocation, because leg lengthening is commonly observed after THA, which results in internal femoral rotation. Combined with internal rotation caused by leg lengthening, excessive reduction in anteversion may increase the risk of anterior impingement and subsequent dislocation. Although combined anteversion provides a valuable theoretical framework [5,6,7], we recommend careful consideration of these biomechanical interactions in clinical practice.

This study had several limitations. First, this study did not include a healthy control group. However, the primary objective of this study was to assess changes in femoral rotation before and after THA and the factors associated with those changes. Additionally, the ethical and practical constraints of performing pelvic-to-knee CT scans in healthy individuals limited the feasibility of such a comparison. Second, we measured the FRA only in the supine position without hip flexion. Internal rotation of the FRA in the supine position may not directly indicate the actual risk of anterior impingement because anterior impingement usually occurs during hip flexion. Cautious interpretation is required when correlating our morphological findings with the clinical risks of dislocation or anterior impingement. Third, the relatively small number of male patients (*n* = 15) limited the feasibility of a robust stratified analysis by sex. Future studies with a more balanced sex distribution are warranted to further clarify the influence of sex on the FRA.

Furthermore, given the extremely low dislocation rate with current computer-assisted THA techniques [28]—with no dislocations observed during our study period—the direct impact of the FRA on dislocation could not be definitively assessed. Finally, our postoperative CT scans were performed between one and four months after surgery, a period when soft tissue healing and muscle recovery are still ongoing. Consequently, the measured FRA may be influenced by transient postoperative factors rather than representing long-term stable alignment. Future large-scale and extended follow-up studies are necessary to confirm the stability of these findings.

## 5. Conclusions

This study demonstrated a significant increase in femoral internal rotation following THA. An increase in anteversion and a decrease in global offset strongly influenced internal rotation in the FRA.

## Figures and Tables

**Table 1:** Patient demographic and surgical data.

Characteristic	
Age (years)	64 ± 10 (34–90)
Sex (women/men)	93/15
Body mass index (kg/m^2^)	24.2 ± 4.5 (14.8–35.9)
Diagnosis (OA/ONFH/RDC or fracture)	97/8/3
Resection of the ISFL (hips)	80 (74.1%)
Resection of the ISFL and conjoint tendon (hips)	34 (31.5%)
Acetabular cup (hips)	
G7 (Zimmer Biomet)	108 (100%)
Femoral stem (hips)	
Avenir complete (Zimmer Biomet)	74 (68.5%)
Taperloc complete full-length (Zimmer Biomet)	34 (31.5%)

Results are expressed as means ± standard deviation (range), unless otherwise indicated. OA, osteoarthritis; ONFH, osteonecrosis of the femoral head; RDC, rapid destructive coxitis; ISFL, ischiofemoral ligament. Zimmer Biomet, Warsaw, Indiana.

**Table 2:** Multivariate linear regression analysis for preoperative to postoperative FRA differences.

	Model 1 ^a^			Model 2 ^b^			Model 3 ^c^		
Explanatory Factors	β	(95%CI)	*p*	β	(95%CI)	*p*	β	(95%CI)	*p*
Anteversion change (°)	−0.37	(−0.57, −0.17)	<0.001	−0.37	(−0.57, −0.17)	<0.001	−0.41	(−0.61, −0.21)	<0.001
Leg lengthening (mm)	−0.28	(−0.70, 0.14)	0.188	−0.34	(−0.76, 0.09)	0.121	−0.35	(−0.74, 0.05)	0.089
Global offset change (mm)	0.31	(−0.04, 0.67)	0.083	0.35	(−0.01, 0.70)	0.058	0.40	(0.06, 0.74)	0.022
Resection of ISFL (reference: preservation of ISFL and conjoint tendon)	−0.32	(−4.99, 4.35)	0.892	−0.65	(−5.48, 4.18)	0.790	0.23	(−4.22, 4.69)	0.917
Resection of ISFL & conjoint tendon (reference: preservation of ISFL and conjoint tendon)	2.17	(−2.89, 7.23)	0.397	2.18	(−3.31, 7.66)	0.433	0.81	(−4.25, 5.88)	0.750

FRA, femoral rotation angle; β, regression coefficients; 95%CI, 95% confidence interval; ISFL, ischiofemoral ligament. ^a^. Adjusted for age and sex. ^b^. Adjusted for age, sex, body mass index and stem variation. ^c^. Model with simultaneous inputs of age, sex, body mass index, stem variation and all explanatory factors in the table.

## Data Availability

The data presented in this study are available on request from the corresponding author due to privacy or ethical restrictions.

## Supplementary Materials

The following supporting information can be downloaded at https://www.mdpi.com/article/10.3390/jcm15072729/s1, Supplementary Table S1: Pre- and Postoperative Intra- and Interobserver Intraclass Correlation Coefficients for Femoral Rotation Angle.

## Abbreviations

The following abbreviations are used in this manuscript:

ALSA	anterolateral supine approach
BMI	body mass index
CT	computed tomography
FRA	femoral rotation angle
ISFL	ischiofemoral ligament
PCL	posterior condylar line
THA	total hip arthroplasty
