# Supplementary material for: Postoperative Changes in Femoral Rotation Angle and Their Influencing Factors Following Total Hip Arthroplasty via Single Approach: A Retrospective CT-Based Study
Source: J Clin Med. 2026 Apr 4;15(7):2729. doi: 10.3390/jcm15072729 (PMC13073369; doi:10.3390/jcm15072729)
Supplement: Supplementary file 1 [file jcm-15-02729-s001.zip › jcm-4195286-supplementary.pdf]

Supplementary Table S1. Pre- and Postoperative Intra- and Interobserver Intraclass Correlation Coefficients for Femoral Rotation Angle

	Preoperative (95%CI)	Postoperative (95%CI)
Intraobserver ICC	0.997 (0.991, 0.999)	0.998 (0.994, 0.999)
Interobserver ICC	0.994 (0.982, 0.998)	0.992 (0.975, 0.998)

ICC; intraclass correlation coefficients, 95%CI; 95% confidence interval
